# Supplementary material for: Understanding drivers of neonatal mortality in Zimbabwe: A machine learning approach using survey data
Source: PLOS Glob Public Health. 2026 Jan 29;6(1):e0004385. doi: 10.1371/journal.pgph.0004385 (PMC12854431; doi:10.1371/journal.pgph.0004385)
Supplement: S1 Fig — (DOCX) [file pgph.0004385.s001.docx]

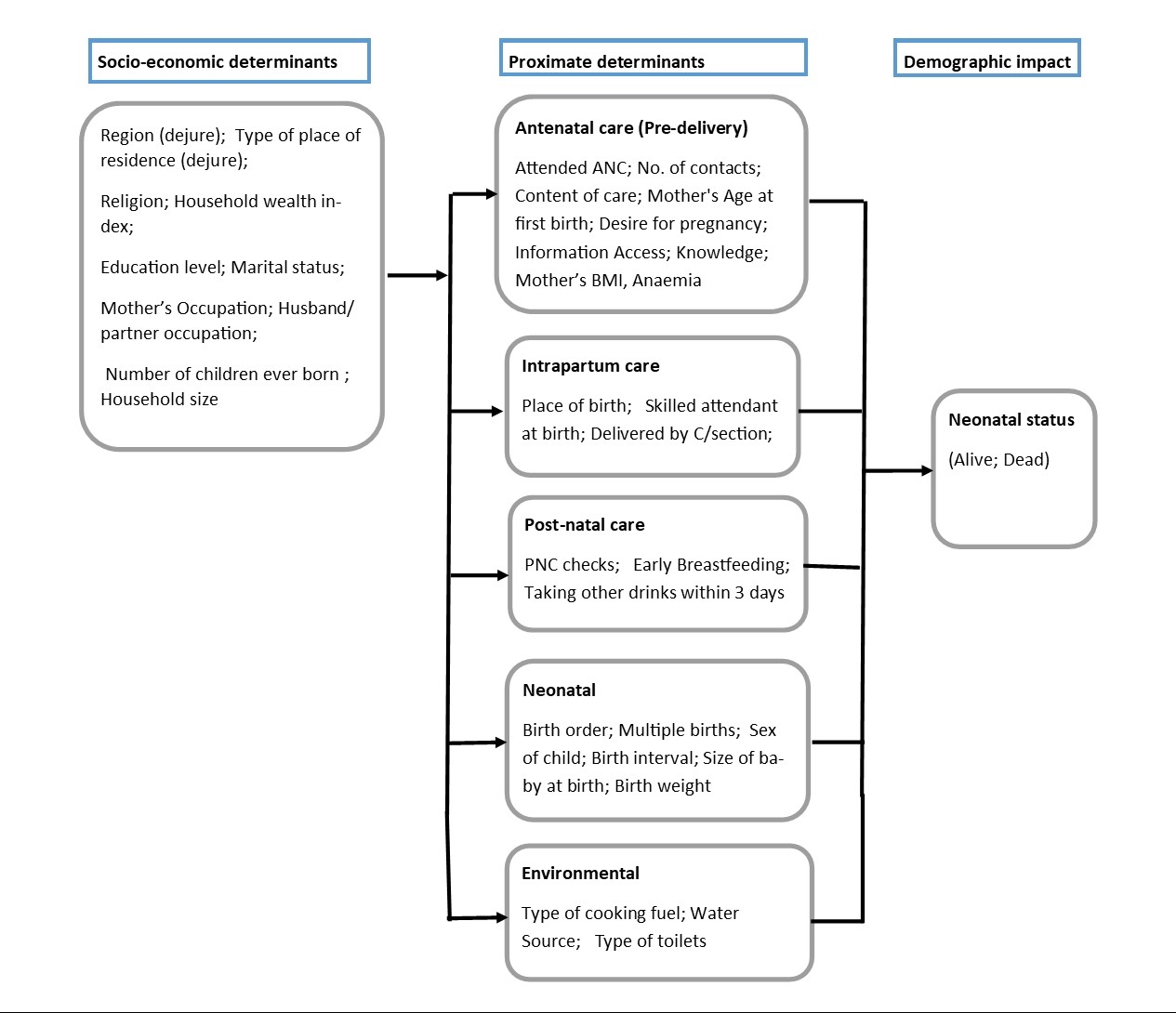


S1 Fig. Conceptual framework for socio-economic, antenatal, intrapartum, and postnatal care factors
