# Supplementary material for: Understanding drivers of neonatal mortality in Zimbabwe: A machine learning approach using survey data
Source: PLOS Glob Public Health. 2026 Jan 29;6(1):e0004385. doi: 10.1371/journal.pgph.0004385 (PMC12854431; doi:10.1371/journal.pgph.0004385)
Supplement: S1 Table — (DOCX) [file pgph.0004385.s002.docx]

S1 Table: Socio-economic, demographic, antenatal, intrapartum, and postnatal care variable types, definitions, and categories

| Variable (variable name in database) | Variable type/scale | Definition and categories |
| --- | --- | --- |
| Socio-economic and demographic | | |
| Survey year (V007) | Nominal | The year of the interview or survey was conducted (2005/2006, 2010/11, 2015) |
| Region (V024) | Nominal | Province of usual residence  ( 1= manicaland, 2 = mashonaland central, 3= mashonaland east , 4= mashonaland west , 5= matabeleland north, 6= matabeleland south, 7 = midlands, 8= masvingo, 9= harare 10 =bulawayo) |
| Type of place of residence (V025) | Nominal | Type of place of residence  (1 = urban, 2= rural) |
| Religion (V130) | Nominal | Religion (1= traditional, 2= roman catholic, 3= protestant, 4= pentecostal, 5= apostolic sect, 6= other christian, 7= muslim, 8= none, 96= other) |
| Age of mother at first birth (V212) | Continuous | Age of respondent at first birth (positive integer) |
| Education level of mother (V106) | Ordinal | Highest education level attended.  (0= no education, 1 = primary, 2= secondary, 3= higher) |
| Household wealth index (V190) | Ordinal | Wealth index quintile urban/rural  1= poorest, 2=poorer 3= middle, 4= rich, 5= richest |
| Marital status (V502) | Nominal | Whether the respondent is currently, formerly, or never married or lived with a partner. (0= never in union, 1= currently in union/living with a man, 2= formerly in union/living with a man) |
| Occupation of mother (V717) | Nominal | 0 = did not work, 1= professional/technical/managerial, 2= clerical, 3= sales, 4= agricultural - self-employed, 5= agricultural – employee, 6= household and domestic, 7= services, 8= skilled manual, 9= unskilled manual; 96= other; 98= don't know |
| Number of children ever born (V201) | Discrete | Total number of children ever born (non-negative integer) |
| Household size (v136) | Discrete | Total number of members in the household (non-negative integer |
| Desire for pregnancy/child wanted (M10) | Nominal | Intention to become pregnant (1=wanted then, 2=wanted later, 3=wanted no more) |
| Antenatal care (Pre- Delivery) | | |
| ANC visits category (derived from M14) | Discrete | Category of antenatal visits during the pregnancy (0= No ANC, 1 =1 to 3 visits, 2= 4+ ANC visits) |
| Tetanus toxoid for mother (M1, M1A, M1D, M1E) | Dichotomous | Fully protected for tetanus (1= yes, 0= no) |
| Blood pressure (M42C) | Dichotomous | During pregnancy - blood pressure taken (1= yes, 0= no) |
| Urine sample taken (M42D) | Dichotomous | During pregnancy - urine sample taken (1= yes, 0= no) |
| Iron supplementation (M45) | Dichotomous | During pregnancy, given or bought iron tablets/syrup (1= yes, 0= no) |
| ANC by health professional (M2A, M2B) | Dichotomous | Health professional provided ANC care (1= yes, 0= no) |
| Intrapartum (Labour & Delivery) | | |
| Place of birth (M15) | Dichotomous | Place of delivery of the child (1= home, 2= health facility, 3= other) |
| Skilled attendant at birth (M3A: M3B) | Dichotomous | Health professional assisted with the delivery of the child (1= yes, 0= no) |
| Delivered by C/section (M17) | Dichotomous | Whether a child was born by caesarian section (1= yes, 2= no) |
| Neonatal |  |  |
| Birth order (BORD) | Continuous | Birth order number gives the order in which the children were born |
| Multiple births (B0) | Ordinal | Twin code gives an order number for each child of a multiple birth (0= single birth, 1= 1st of multiple, 2= 2^nd^ of multiple, 3= 3^rd^ of multiple, 4= 4^th^ of multiple, 5= 5^th^ of multiple) |
| Sex of child (B4) | Dichotomous | Sex of child (1= male, 2= female) |
| Birth interval (B11) | Dichotomous | Preceding birth interval (< 24 months (short), 24 – 59 months (normal), 60 months + (long), first birth) |
| Size of baby at birth (M18) | Ordinal | Size of child as reported subjectively by the respondent (1= very large, 2= larger than average, 3= average, 4= smaller than average, 5= very small, 8= don’t know) |
| Birth weight (M19) | Continuous | Weight of child at birth given in grams |
| Post-natal care |  |  |
| Early Breastfeeding (M34) | Dichotomous | Time after the birth at which the respondent first breastfed the child (0= immediately, 1= >1 hour) |
| Newborn postnatal care (M70) | Dichotomous | Received postnatal care within 2 months (1 = yes, 0 = no) |
| Outcome variable |  |  |
| Neonatal death (derived from B5 and B7) | Binary | Death of child within a month. Derived from (B7) Age at death of the child in completed months gives a calculated age at death from the reported information (1 = yes = age at death ≤ 1 month, 0 = no = age at death > 1 month or Child is Alive) |
