# Supplementary material for: Understanding drivers of neonatal mortality in Zimbabwe: A machine learning approach using survey data
Source: PLOS Glob Public Health. 2026 Jan 29;6(1):e0004385. doi: 10.1371/journal.pgph.0004385 (PMC12854431; doi:10.1371/journal.pgph.0004385)
Supplement: S2 Table — (DOCX) [file pgph.0004385.s003.docx]

S2 Table: Variables with missing values and percentage of missing values (N= 16,941)

| Variable | Missing (n) | Percentage missing (%) |
| --- | --- | --- |
| Child protected against tetanus | 3,650 | 22% |
| ANC by a skilled provider | 3,643 | 22% |
| ANC visit | 3,647 | 22% |
| ANC_visit category | 3,647 | 22% |
| ANC_timing | 4,529 | 27% |
| BP check in ANC | 4,529 | 27% |
| Urine check in ANC | 4,530 | 27% |
| Iron supplementation | 3,650 | 22% |
| Birth weight | 4,159 | 25% |
| Newborn postnatal care | 7,897 | 47% |
