# Supplementary material for: Understanding drivers of neonatal mortality in Zimbabwe: A machine learning approach using survey data
Source: PLOS Glob Public Health. 2026 Jan 29;6(1):e0004385. doi: 10.1371/journal.pgph.0004385 (PMC12854431; doi:10.1371/journal.pgph.0004385)
Supplement: Attachment — Submitted filename: Response to Reviewers_19.02.2025.docx [file pgph.0004385.s004.docx]

­Absolom Mbinda

Countdown 2030 Fellow

Department of Global, Public Health and Family Medicine,

University of Zimbabwe, Zimbabwe

February 12, 2025

Academic Editor,

PLOS Global Public Health

Dear Mufaro Kanyangarara

RE: Manuscript PGPH-D-24-02248 response to reviewers.

Thank you for allowing us to submit a revised draft of the manuscript “Understanding drivers of neonatal mortality in Zimbabwe: A machine learning approach using survey data” for publication in *PLOS Global Public Health Journal.* We are grateful for the valuable comments that the reviewers provided to enhance the quality of our manuscript. We would also like to appreciate the time and effort that you and the reviewers committed to providing this feedback. Most of the suggestions that were made by reviewers have been incorporated and the changes are highlighted in the manuscript with track changes. Below, we have prepared a point-by-point response, in blue, to each reviewer’s comments or concerns. The line or page numbers refer to the revised manuscript in tracked changes.

**Reviewer #1:**

Zimbabwe’s neonatal mortality rate (NMR) remains high, off track to meet the 2030 SDG targets, and unlike other child mortality indicators that have improved, NMR has remained stagnant. This study uses machine learning to identify key predictors contributing to neonatal mortality in Zimbabwe, analyzing pooled data from the 2005-2015 Zimbabwe Demographic and Health Survey (ZDHS). Based on sensitivity, and Area Under the Receiver Operating Curve (AUC ROC), Gradient Boosting Model (GBM) was selected as the top-performing model for predicting neonatal mortality. Using this model Birth weight, household size, and birth order were identified as significant predictors while logistic regression showed that lower birth weight and higher birth order increased neonatal mortality risk, while larger household size had an inverse relationship.

This study offers valuable insights into the predictors of neonatal mortality, holding particular significance as the first study in Zimbabwe to utilize machine learning for identifying these factors. However, to enhance clarity and readability, the manuscript would benefit from grammar revision and edits. This includes breaking up lengthy paragraphs, ensuring correct tense usage, refining phrasing, and incorporating appropriate punctuation. Additionally, below are my specific feedback on each section to consider for further refinement.

**Authors’ response:** Thank you very much for your comments and for taking the time to review.

The introduction effectively presents the global and regional context of neonatal mortality, narrowing down to Zimbabwe’s specific challenges in Sub-Saharan Africa. The authors clearly highlight the role of ML and need (identified gap) for this study.

**Authors’ response:** Thank you very much!

The Methods is well-organized and clearly explains the model selection.

**Authors’ response:** Thank you very much!

The result section is well crated. Tables and figures were used when appropriate. The authors also have done an excellent job of comparing outputs from both linear regression and machine learning models. This approach adds depth to the analysis and helps understand similarities and distinction between these methods. and demonstrating how they complement each other.

**Authors’ response:** Thank you very much!

The Discussion section provides valuable insights and compares the results well with existing literature. However, some of the statements should be strengthened by incorporating proper references (citations) to enhance the credibility and provide a clearer basis for drawing conclusions based on the findings.

**Authors’ response:** Additional references have been put in the discussion section as suggested.

Strengths and Limitations section effectively highlights the study's strong points and acknowledges its constraints particularly with respect to the data set and machine learning approach. Adding limitations with using the specific GBM model applied would make it more contextual for interpreting the findings.

**Authors’ response:** We have added specific strengths and limitations of the GBM model on top of the ones that are common to all ML models on lines 452 - 455.

The conclusion effectively summarizes the role of machine learning in identifying predictors for neonatal mortality. However, I would suggest revising phrases like "I argue" in favor of a unified authorial voice (using “we”) ensuring the recommendations reflect a team consensus. In cases where consensus has not been achieved, this should be indicated. Including references to support proposed argument/strategies would also enhance the credibility and feasibility of the recommendations.

**Authors’ response:** Thank you, we have revised line 470 as suggested.

Additionally, for future studies, it would be valuable to explore the specific causes of the risk factors identified and investigate causal relationships or interactions between variables. This could yield more comprehensive insights into the underlying dynamics driving neonatal mortality, allowing for the design of more targeted and effective interventions.

**Authors’ response:** We have added as suggested on lines 486 - 489.

Overall, this manuscript offers timely and valuable insights into the predictors of neonatal mortality using machine learning in the country. The findings provide a strong foundation for future research and could inform targeted interventions to reduce neonatal mortality rates (NMR). However, the manuscript would benefit from grammar revisions and a more precise use of citations/references to align with publication standards. Incorporating this and the above-mentioned suggestions would further enhance clarity, and overall quality of the manuscript.

**Authors’ response:** Thank you, we have incorporated the suggested changes.

**Reviewer #2:**

Thank you for the opportunity to review this article
The manuscript is generally well-written. However, I have a few concerns that should be addressed before publication.

**Authors’ response:** Thank you very much for the comments and for taking the time to review.

Introduction section
I recommend modifying lines 50 to 52 as follows, “Neonatal mortality rate (NMR) has almost halved, decreasing from 37 to 18 neonatal deaths per 1,000 live births between 1990 and 2021 globally. However, variations persist across geographic regions and countries (1,2).” Instead of “Neonatal mortality rate (NMR) has almost halved from 37 to 18 neonatal deaths per 1,000 live births between 1990 and 2021 globally but there remain variations across geographic regions and countries (1,2).”

**Authors’ response:** The lines have been modified as suggested.

Line 53 and 54 should be modified to make the sentence clear and easy to comprehend.

**Authors’ response:** The lines have been modified, and the revised text reads “Zimbabwe is one of the countries in sub-Saharan Africa (SSA) region with an NMR (32 neonatal deaths per 1,000 live births) that is higher than regional average”.

Results
Line 306 should be written as “Two variables, birth order (aOR [95%CI]; 1.29 [1.14 - 1.45]) and birth weight (aOR [95%CI]; 0.99 [ 0.998 – 0.999]) were significant in logistic regression.” Instead of “Two variables, birth order (aOR [95%CI]; 1.29 [1.14 - 1.45]), birth weight (aOR [95%CI] and 0.99 [ 0.998 – 0.999]) were significant in logistic regression.”

**Authors’ response:** The line has been updated as suggested, the update is found on lines 315 -316.

Discussion
From lines 357 to 361, why were the reasons for the birth order result not stated/explained in the discussion, unlike those for birth weight and birth interval? I recommend doing so.
Why was ‘birth interval’ discussed as a predictor in the discussion but was not included in the conclusion?

**Authors’ response:** We have added the reasons for birth order on lines 367 – 369. We have added the birth interval in the conclusion on line 467. We had just highlighted the top 5 predictors initially.

Answers
The above grammatical errors should be corrected to ensure clarity and easy comprehension of the article.

**Authors’ response:** The errors have been corrected as highlighted above.

Reasons should be stated for each findings in the study with references from other articles to support claim. Findings should not just be stated in the study.

**Authors’ response:** Thank you, we have added the reasons.

If birth interval was found to be a predictor, it should be included in the conclusion after discussion.

**Authors’ response:** We have added birth interval in the conclusion on line 467. We had just highlighted the top 5 predictors initially.

Congratulations to the entire team for this study.

**Authors’ response:** Thank you very much!

**Reviewer #3**:

Abstract: It would be better if you write a structured abstract that includes introduction, objective, material & method, Results and conclusion, in that order.

**Authors’ response:** Thank you very much for the comment, the abstract is structured with introduction, objective, material and methods, results and conclusion in that order but without those clear subheadings. This is as per guidelines from the journal. We are happy to put those headings if the Editor suggests so.

Data sharing: Publish the data related to your analysis

**Authors’ response:** Thank you, the following data availability statement was shared as part of the manuscript’s initial submission. The study was conducted using publicly available data from the Demographic Health survey Program repository (<https://dhsprogram.com/data/dataset_admin/index.cfm>). After registering an online account with DHS and submitting an online application stating the research question, access to relevant datasets on the DHS repository was granted.

NB: Generally, a very good study

**Authors’ response:** Thank you very much!

**Reviewer #4:**

Introduction
Lines 57, 57 “NMR has stagnated in Zimbabwe over the past two decades while all other under 5 and childhood mortalities have been declining.”
This information needs to be verified further using local data sources such as DHIS. NMR appear to be increasing in Zimbabwe since 2019.

**Authors’ response:** Thank you for your comment, we have revised the sentence from lines 56 -58 to read, “NMR has increased by 10% in Zimbabwe in the two decades between 1999 and 2019 while all other under 5 and childhood mortalities have been declining according to national surveys”.

Method
Line 107. “The outcome variable was neonatal death (yes/no)”
How did you arrive at yes/no (mortality or otherwise) from the data sets?

**Authors’ response:** We have added additional text to explain further from lines 110 -115. A neonatal death was counted as a death that occurred within the 1st month (≤ 30 days). This was derived from variable B7 (age at death of the child in completed months) in the dataset which gives a calculated age at death from the reported information.

Line 108. “While the independent variables were selected based on adaptation of the Mosley and Chen framework and other similarly adapted frameworks…”
It is probably clearer to list/name adapted sources rather than just providing reference.

**Authors’ response:** We have revised the sentence and listed the adapted sources as indicated in lines 109 -110.

Lines 121 to 123. “The modeling process was done in systematic steps which involved 120 exploratory data analysis, data preparation, model building which involved splitting data into train and test, cross validation, down sampling to address imbalances, hyperparameter tuning for selected models to avoid overfitting”
Entire paragraph sounds theoretical. Authors should perhaps describe how you arrived at the model used without re-stating theoretical phrases.

**Authors’ response:** Thank you for the comment, this was an introductory statement for this sub-section, and we have added some text reflected on line 129 which highlights that the steps are described in the follow-up text in relation to our study to emphasize what we did.

Line 131. “Merged births dataset was filtered by age of child (< 60 months) at time of survey before exploratory data analysis (EDA) was done”
What was the rationale for a merger and why was there the need to filter? DHS data across the rounds are supposed to be identical. This is not clear to me.
**Authors’ response:** The birth datasets from three different rounds of ZDHS were downloaded separately and were combined (merged) to make one dataset which was used for analysis. We have modified the text to make this a bit clearer in the revised manuscript from lines 134 -135. We have added text from lines 138 -140 on the need to filter the dataset by < 60 months as this was our unit of analysis and the births whose birth history contained information on variables of interest. The DHS collects and records birth data on all children ever born to women of reproductive health at the time of the survey and some questions are not asked for births of 60+ months due to a long recall period e.g. antenatal and postnatal care.

Outcome variable
Although the authors stated that neonatal mortality was the outcome of interest as binary, this variable does not appear anywhere in the model/Table. Your main variable need to come clear and reported in the Table.

**Authors’ response:** Thank you for the comment, we think that Table 2 from Line 209 shows the outcome variable distribution by survey year. We have also added a row on totals on the same table.

Results
Line 36 “while household size had an inverse relationship with neonatal mortality (aOR [95%CI]: 0.87 [0.79 – 0.95]).”
1. Do you mean with increased household size, there is a corresponding decrease in neonatal mortality? In that case why is your recommendation advocating for the use of family planning to regulate household size?

**Authors’ response:** We have revised line 36 to read “negatively associated with odds of neonatal death” and have removed household size on the recommendation of family planning on line 40.

Table 5. “Household size (v136) 0.87 [0.79 – 0.95] <0.001*”
Is this variable “household size” continuous variable? In any case the interpretation of the results could is problematic despite the statistically significant p-value. You are suggesting that increased household size led to decrease neonatal mortality, but your outcome variable is binary (yes/no) and this is tricky because your finding suggest that an increase of 1 unit in family size lead to a decrease of 13% neonatal mortality. Your outcome of interest is binary (yes/no) dead or alive which is why you chose logistics regression. I don’t see how you can explain the influence of household size in the manner that you did i.e. inversely related. You probably need to drop the household variable or categorize it to allow for a meaningful interpretation to avoid misleading readers.

**Authors’ response:** Thank you for your comment, the variable household size is numerical (integer), and we believe the logistics regression model allows for both continuous and categorical variables as independent variables. We have modified the explanation of the results in line 36 and lines 394-396 to make this clearer in line with how results of continuous independent variables are interpreted in a logistics regression model and have added “negatively associated with the odds of neonatal death” instead of inversely related to neonatal death. The revised interpretation of this would be, “for a unit increase in household size, the odds of neonatal death decreases by 13%” instead of, “for a unit increase in household size neonatal mortality decreases by 13%”. Although this finding is unexpected, it corroborates with another unexpected finding in similar ML study as highlighted in discussion section from line 393.

Table 5. “Age of mother at first birth (V212) 0.99 [ 0.94 – 1.06] 0.950 29.45”
What are your criteria for determining predictors and why does “Age of mother” matter as capture in concluding discussion? Age is not categorized, and not statistically significant. How did you arrive at its relevance as a predictor?
**Authors’ response:** Thank you for the comment, we employed the ML approaches to determine the predictors and logistics regression to understand the effect direction for the predictors. ALL predictors with a relative importance score above zero were important using ML approaches while those with a p-value <0.05 were significant using logistics regression. Table 5 shows both results of ML and logistics regression for all predictors that were important using logistics ML methods. The primary approach in this study is ML and age matters as it’s the 4^th^ most relatively important variable as shown in Fig 2 from line 283. Also, we believe the logistics model can take continuous and categorical variables as independent variables and it’s a matter of how the results are interpreted. While age was not significant in logistics regression, it was one of the most relatively important predictors using the ML approach. This is the reason why we highlighted in the discussion that we may need to have the classical/traditional statistical approaches and ML complement each other instead of relying on one approach as ML may uncover complex relations that we may fail to get using traditional methods. We have modified the font on the variable Age in Table 5 from bold to normal to add clarity.

Discussion
Line 340. “Birth weight is a function of maternal nutrition, health status and care during pregnancy”
I’m not sure if the maternal nutrition, and health status are mutually exclusive from care during pregnancy. Outcome of functions over lapses. Sentence should be re-phrased.
**Authors’ response:** We have re-phrased the sentence to read, “Birth weight is a function of overall pre-conception maternal nutrition and health status and care given during pregnancy” on line 348-349.

Overall
Useful study that would contribute to literature and drive policy development in support of the fight against neonatal mortality in Zimbabwe.

**Authors’ response:** Thank you very much!
