# Supplementary material for: Understanding drivers of neonatal mortality in Zimbabwe: A machine learning approach using survey data
Source: PLOS Glob Public Health. 2026 Jan 29;6(1):e0004385. doi: 10.1371/journal.pgph.0004385 (PMC12854431; doi:10.1371/journal.pgph.0004385)
Supplement: Attachment — Submitted filename: Response to Reviewers_PGPH_25.09.2025.docx [file pgph.0004385.s005.docx]

­Absolom Mbinda

Countdown 2030 Fellow

Department of Global, Public Health and Family Medicine,

University of Zimbabwe, Zimbabwe

September 23, 2025

Staff Editor,

PLOS Global Public Health

Dear Joanna Tindall

RE: Manuscript PGPH-D-24-02248 response to reviewers.

Thank you for allowing us to submit a revised draft of the manuscript “Understanding drivers of neonatal mortality in Zimbabwe: A machine learning approach using survey data” for publication in *PLOS Global Public Health Journal.* We are grateful for the valuable comments that the reviewers provided to enhance the quality of our manuscript. We would also like to appreciate the time and effort that you and the reviewers committed to providing this feedback. Most of the suggestions that were made by reviewers have been incorporated, and the changes are highlighted in the manuscript with track changes. Below, we have prepared a point-by-point response, in blue, to each reviewer’s comments or concerns. The line or page numbers refer to the revised manuscript in tracked changes.

Reviewer #1: The authors have addressed the feedback provided. Congratulations to the team!

**Authors’ response:** Thank you very much!

Reviewer #2: (No Response)

**Authors’ response:** Thank you very much!

Reviewer #3: Thank you for addressing all the comments

**Authors’ response:** Thank you very much!

Reviewer #4: All three round of ZDHS are somewhat old and do not exactly reflect the current situation in Zimbabwe. This is deem a weakness and should be reflected in the discussion and limitation. The paper has improved greatly and I have no further comments to make.

Congratulations to the authors.

**Authors’ response:** Thank you very much!

**Reviewer #5:**

Dear Authors
Please refer to my comments below:
1. Line 54 - please state the regional average and add a reference

**Authors’ response:** The regional average for sub-Saharan Africa (SSA) was added together with reference as suggested on lines 56-57.

2. Line 57-59 - if the contribution of NMR to U5MR is increasing, is the UFMR still declining?

**Authors’ response:** During the period under study, all under-five and childhood mortalities declined, as highlighted in lines 60-61, while only NMR increased, and this led to the increase in percentage contribution to under-five mortality.

3. Line 69 - please spell out ML on the first instance here

**Authors’ response:** This has been added on line 72.

4. Line 119 - no of ANC visits?

**Authors’ response:** Have revised sentence on line 121 to read “number of ANC visits”.

5. Line 119 - what timepoint was BP and urine testing done? Is this based on maternal recall or medical records?

**Authors’ response:** The BP and urine testing questions were based on maternal recall and whether at least a BP or urine test was done during ANC without specific reference to any time point. We have edited line 122 to indicate testing at least once during ANC.

6. Line 143 - Was imputation attempted to address missingness?

**Authors’ response:** We conducted the imputation of missing data, lines 148 -150.

7. Why werent F1scores reported considering the imbalanced dataset?

**Authors’ response:** Thank you very much. F1 scores were reported on lines 270 -271.

8. Line 217 - Not sure what impact a urine sample being obtained has on neonatal mortality?

**Authors’ response:** Urine testing is one of the key components of quality ANC services. Urine testing, while not directly linked to neonatal mortality, is important in screening for infections and other conditions such as pre-eclampsia, which may cause adverse neonatal outcomes. Failure to get screened reflects on the quality of ANC services.

9. Line 219 - Would be good to show how many were LBW

**Authors’ response:** This has been added to Table 3.

10. Table 3 - what does wanted no more indicate?

**Authors’ response:** This indicates women who wanted to stop childbearing and had unwanted children, i.e., wanted no more children. This is a standard demographic health survey response option.

11. Why was birth interval > 48 months? The usual practice is 24 months

**Authors’ response:** Thank you very much! We have adjusted the analysis to include <24 months (short birth interval), 24 -59 months (normal), and 60+ months (long) based on further literature review.

12. Considering the predictive factors identified through the ML process, I dont see what additional evidence has been generated through this work.

**Authors’ response:** To the best of our knowledge, this study is the first in Zimbabwe that has used ML approaches to develop prediction models and identify neonatal mortality predictors using nationally representative survey data over a longer period of time. Previous studies were localized to sub-regions of the country or hospitals and were not nationally representative, and employed traditional methods, while our study employed both ML and traditional methods. While some predictors are known at a global or regional level, we believe this study is useful in contextualizing the predictors to Zimbabwe, as well as ranking the features identified in terms of importance.

**Reviewer #6**:

Based on a thorough scientific review of the manuscript titled "Understanding drivers of neonatal mortality in Zimbabwe: A machine learning approach using survey data", here are detailed and scientifically grounded comments supporting rejection of the paper in its current form:

1.The machine learning models perform poorly (AUC ≤ 0.72; precision & F1 < 0.05). Such low performance limits practical utility and undermines the model's predictive value.

**Authors’ response:** Thank you very much! The study’s primary goal was exploratory—to identify potentially important predictors—rather than to construct a clinically deployable prediction tool. This could inform such development in the future.

2.The extreme class imbalance (74:1) was managed only by down sampling, leading to potential information loss. Better methods (e.g., SMOTE, cost-sensitive learning) were not used or discussed.

**Authors’ response:** Thank you very much! We considered this important comment and implemented down-sampling, up-sampling, and SMOTE in the revised manuscript and chose the method that produced the best results for our data.

3. The study adds little beyond traditional methods. Predictors identified by ML are mostly known and show minimal novelty. Variable importance was misinterpreted as causal, without robust validation.

**Authors’ response:** Thank you very much! To the best of our knowledge, this study is the first in Zimbabwe that has used ML approaches to develop prediction models and identify neonatal mortality predictors using nationally representative survey data over a longer period of time. Previous studies were localized to sub-regions of the country or hospitals and not nationally representative, and employed traditional methods, while our study employed both ML and traditional methods. While some predictors are known at a global or regional level, we believe this study is useful in contextualizing the predictors to Zimbabwe, as well as ranking the features identified in terms of relative importance. We have edited language like causes of neonatal mortality to predictors of neonatal mortality or factors associated with neonatal mortality in the results and discussion so that we don’t appear to infer causality. The primary intent was to identify relatively predictive variables, not to draw causal inferences.

4. No external or temporal validation was conducted. Results are based solely on internal cross-validation, limiting generalizability.

**Authors’ response:** Thank you very much! The study’s primary goal was exploratory, that is, to identify potentially important predictors rather than to construct a clinically deployable prediction tool. What we have done could inform such development, considering our recommendations, such as utilizing electronic health records as a data source. In developing the models, we followed all the key steps which included setting aside a testing sample (70% - 30% train -test split) to validate our results. The testing sample, which was kept separate from the training dataset, was used to evaluate the performance of the models and gauge how they could fare in real-world situations (generalizability to unseen data). Considering that the primary intent was exploratory, to identify potentially important predictors rather than to construct a clinically deployable prediction tool, we believe there was no need for a third dataset to do external validation.

5. Important variables were dropped due to missingness. No imputation or sensitivity analysis was performed. Confounding and bias remain unaddressed.

**Authors’ response:** Thank you very much! We have revised our manuscript and conducted imputation of missing variables, lines 148 -150, to ensure some variables were not dropped and also address bias due to missingness and the dropping of some variables. In logistic regression, we put all the variables considered for modeling, including those that were not selected by the Boruta algorithm for modeling, to further address confounding and bias.

5. The manuscript makes causal policy recommendations from purely associative, cross-sectional data without using causal inference methods.

**Authors’ response:** Thank you very much! We have tried to indicate and include in our narratives that the predictors we identified in our study are factors associated with neonatal mortality, not those that cause neonatal mortality. Our conclusions and recommendations, we believe, are based on the strength of associations identified. We are happy to correct any specific lines that you think are not appropriate.
