# Supplementary material for: Understanding drivers of neonatal mortality in Zimbabwe: A machine learning approach using survey data
Source: PLOS Glob Public Health. 2026 Jan 29;6(1):e0004385. doi: 10.1371/journal.pgph.0004385 (PMC12854431; doi:10.1371/journal.pgph.0004385)
Supplement: Attachment — Submitted filename: Response to Reviewers_PGPH_22.12.2025.docx [file pgph.0004385.s006.docx]

Absolom Mbinda

Countdown 2030 Fellow

Department of Global, Public Health and Family Medicine,

University of Zimbabwe, Zimbabwe

December 22, 2025

Staff Editor,

PLOS Global Public Health

Dear Dr. Rufaro Kanyangara

RE: Manuscript PGPH-D-24-02248 response to reviewers.

Thank you for allowing us to submit a revised draft of the manuscript “Understanding drivers of neonatal mortality in Zimbabwe: A machine learning approach using survey data” for publication in *PLOS Global Public Health Journal.* We are grateful for the valuable comments that the reviewers provided to enhance the quality of our manuscript. We would also like to appreciate the time and effort that you and the reviewers committed to providing this feedback. Most of the suggestions that were made by reviewers have been incorporated, and the changes are highlighted in the manuscript with track changes. Below, we have prepared a point-by-point response, in blue, to each reviewer’s comments or concerns. The line or page numbers refer to the revised manuscript in tracked changes.

EDITOR:

**Major Comments**

1. While PLOS Global Public Health accepts manuscripts of any length, a 8,000-word paper is excessive. The authors are encouraged to present and discuss their findings more concisely.

**Authors’ response:** Thank you very much! We have tried to cut down on some text in the results and discussion section. We have done a word count of the manuscript body excluding the abstract, tables, references, and supplementary material, and the word count is roughly 6,373 ( Introduction – 897, Methods – 1,478, Results – 1,389, Discussion, limitations, and conclusion – 2,609). We hope this will suffice. If this isn’t enough, we propose to trim down the discussion section to focus on the top five predictors instead of the top ten.

2. Previous reviewers noted that it was unclear what added value the machine learning (ML) approach brings beyond traditional regression. The authors still have not adequately addressed why ML was necessary, how it performed better, and what insights it uncovered that classical methods may have missed. For example, including a short paragraph summarizing existing studies using ML in LMICs to better frame your contribution.

**Authors’ response:** We have added a paragraph from lines 80 – 96 to put our study into a relevant context. The goal in this study was to use ML as the analysis tool, given the gaps identified in the literature, such as its limited use in identifying neonatal mortality factors in developing countries, including Zimbabwe, where it has never been used on the same subject. Additionally, to the best of our knowledge, no study has been done in Zimbabwe using national survey data over a longer period (10 years) to identify predictors of neonatal mortality, whether using ML or traditional methods. All studies done were localized, and some were focused on newborns with specific conditions.

3. The authors mention using unweighted cases (n = 16,941) for modeling and applying survey weights for frequencies only. Why were weights not used in model training or performance evaluation? If the models were trained on unweighted data, statements such as “The results from this study are representative of drivers of neonatal mortality in Zimbabwe” are misleading.

**Authors’ response:** We have removed such statements. There is limited information on the use and implementation of survey weights in ML, especially using the caret package in R. Some algorithms do not account for survey weights. We also looked at the frequency distribution of weighted and unweighted data, and the differences were minimal. Given that this exploratory analysis focused on prediction rather than inference, we believe there would be minimal difference, if any, in the results between weighted and unweighted analysis. We also looked at several similar studies, and they were silent on the implementation of weighted ML analysis using DHS data, although they went on to indicate they used nationally representative survey data. We acknowledge this comment.

4. The authors state that a 70/30 random split was used between training and test sets and that 10-fold cross-validation (repeated three times) was implemented during training. It is unclear whether the split or the cross-validation folds were stratified by neonatal death outcome.

**Authors’ response:** A stratified random split was done based on the neonatal death outcome variable. We have edited line 177 to reflect this.

5. Please clarify whether hyperparameter tuning occurred within an inner cross-validation loop (nested CV). If not, justify the current approach or consider re-running the models using nested CV.

**Authors’ response:** Hyperparameter tuning was done in an inner cross-validation loop, and we have edited line 203 to reflect that.

6. The authors state that a total of 30 out of the 33 variables included in the model-building process were selected by the XGBoost model as relatively important, but do not explain which importance metric was used (gain, cover, or split frequency) or how correlated predictors were handled.

**Authors’ response:** We have added the metric used in line 303. In lines 173 -175, we have added a statement on correlation.

7. It is unclear why Boruta was used if XGBoost was the main model. Also for the Boruta algorithm, specify whether it was applied before or after the train-test split. The authors note that Boruta was used to remove “unimportant variables.” For transparency, please report the number of confirmed, tentative, and rejected features at the end of the Boruta run.

**Authors’ response:** Boruta was applied after the train-test split. We have added text in line 205 on when it was applied and lines 271- 272 on the selected, tentative, and rejected variables.

8. While the language has improved to reduce causal interpretation, several statements still imply causality. Additionally, throughout the manuscript, terms such as drivers, determinants, and key predictors are used interchangeably. “Drivers” and “determinants” imply causality and should be replaced with “predictors” or “associations.”

**Authors’ response:** We have removed drivers and replaced them with predictors in the manuscript body as suggested.

9. Authors state “Receiving newborn postnatal care was found to be protective in this study, and this underscores the need for mothers and their babies to access and utilize appropriate health services soon after delivery (22).” Deaths occurring before postnatal visits may artificially inflate the apparent protective effect of PNC. Because PNC often occurs days after birth, only surviving neonates are “eligible,” leading to selection bias.

**Authors’ response:** According to the Zimbabwe postnatal care guidelines corresponding to the period of the surveys, a newborn is supposed to receive postnatal care starting within 24 hours after birth, not days after birth. Ideally, a mother who delivers at a facility is supposed to be kept for at least 24 hours at the facility, depending on condition and availability of space. Due to space limitations, which are common especially at big facilities, they may be discharged within 6 hours after the 4^th^ stage of labour if clinically stable. Those who deliver at home are supposed to visit the facility immediately, within the first 24 hrs, for the postnatal checks for the baby and the mother. There are subsequent checks done at day 1, 3, day 5, day 7 (for primigravidas only), and 6 weeks at the nearest facility. Our variable was whether newborn PNC was given (at any point after discharge from facility/home delivery, including the first 24 hrs or day 1). In our study, only 68% delivered at the facility, and the others were delivered outside the facility. Those who deliver with caesarean sections, about 5%, are kept in the facility for 3 -5 days, and the majority will be discharged early. This increases the proportion of those eligible for a day 1 or day 3 visit among those delivered at the facility, while all those delivered at home are eligible. Every newborn should receive PNC early, especially within the first 24 - 48 hrs, to increase chances of survival. While there may be some who may die soon after delivery and before discharge, this should not be a huge percentage (limited information available on pre-discharge neonatal mortality). The scenario presented and the earliness of the first 2 visits, make selection bias very minimal. We have added text in the limitations section in lines 525 – 526.

10. “Lower birth weight (aOR [95% CI]: 0.9997 [0.9995–0.9999]) was positively associated with odds of neonatal mortality… interventions should focus on preventing and addressing the effect of birth weight on child survival.”  Birth weight is a strong predictor but not necessarily a modifiable causal factor.

**Authors’ response:** Birth weight is a function of several things, and a multidimensional approach is needed, which includes pre-conception health and nutrition, pregnancy health, and nutrition. Preventative measures can be taken, such as promoting a healthy diet (balanced nutrition) before and during pregnancy, as well as screening and treating illnesses or other conditions before conception and during pregnancy. We believe a healthy expecting mother on a healthy diet can modify the birthweight of the baby. Alternatively, kangaroo mother care is a known cost-effective measure that helps newborns with low birth weight to gain weight and reduces mortality by about 40% for babies born with low birth weight. These interventions can help modify birth weight. Prevention measures should be promoted, and special care should be instituted to improve chances of survival. We have edited the text in line 40.

**Minor Comments**

1. The aOR for birth weight (0.9997) is not intuitive. Please present it on an interpretable scale e.g. per 100 grams

**Authors’ response:** Added text in line 378 as suggested.

2. Given that several variables had >20% missing data, justify the assumption that data were missing at random (MAR).

**Authors’ response:** Thank you very much! We tested the association between the missingness in the variables with missing values and other observed variables. This revealed some associations providing evidence against missing completely at random (MCAR). Also, domain knowledge of the missing variables’ relation to observed ones assisted in coming up with this assumption over other mechanisms. We have edited text in lines 166 -168.

3. It is unclear why all the listed models were included. For instance, why include both GBM and XGBoost, given their similar architecture?

**Authors’ response:** We reviewed the literature and found that there were several models that were implemented in different settings and performed well. We sought to implement a variety of these for our dataset to try to find the best for our data. GBM and XGBoost have similarities but are different. We have edited the text in line 181.

4. There is no mention of random seed setting, which raises concerns about reproducibility not knowing if the code will be shared.

**Authors’ response:** We have added that in the text in line 147, this was always implemented.

5. Given only 473 neonatal deaths, there is a risk of overfitting, consider reporting the events-per-variable (EPV) and discuss overfitting risk.

**Authors’ response:** Have added EPV in line 276.

6. The description of the hybrid SMOTE procedure is confusing. Creating five synthetic minority samples per case and then downsampling the majority class to equal size may produce an artificially balanced dataset that departs from real-world prevalence. Potential ecological validity issues and model calibration (e.g., Platt scaling or probability adjustment) afterward may be needed.

**Authors’ response:** We have updated the description. In data balancing, the goal is to ensure the class distribution in the outcome variable is close to 50% - 50%. Instead of creating too many synthetic samples for each minority case, using the DMWR package, we created only five and then retained the majority cases in such a way that the class distribution of outcome balance was 50% - 50%. You can choose the proportion of majority cases to retain in relation to minority cases, and this is done randomly and is not artificially balanced. We have edited the text to remove hybrid.

7. Given the high class imbalance, the authors should consider using Precision–Recall (PR) curves and AUC-PR, which may be more informative than ROC curves.

**Authors’ response:** Thank you very much, and we acknowledge the comment! We believe once the data is balanced, we can use the ROC curves.

8. The authors report precision and F1-scores around 15%, implying a high false-positive rate. This should be discussed explicitly in the Limitations section.

**Authors’ response:** We have added text in lines 544-545.

9. There was no explicit discussion of multicollinearity. Please report correlation checks or variance inflation factors (VIFs).

**Authors’ response:** We have added text in lines 173 -174.

10. “Support Vector Mechanism (SVM)” should read “Support Vector Machine (SVM),” and “hot encoding” should be “one-hot encoding.”

**Authors’ response:** Edited as suggested.

11. Early breastfeeding and newborn PNC appear protective but may be susceptible to survivor bias and reverse causation. This should be discussed in the Limitations section, and ideally, sensitivity analyses excluding early neonatal deaths or stratified by survey round should be conducted.

**Authors’ response:** Early breastfeeding initiation is breastfeeding within the first hour. We believe that very few babies die in the first few minutes to an hour, although this is possible. Once a mother gives birth, a baby is eligible for breastfeeding from the first minute. Failure to breastfeed early has adverse effects. We have added text in lines 525 – 526.

12. The Limitations section does not discuss potential temporal confounding from pooling data across survey years. Did you adjust for survey year or conduct any sensitivity analyses by survey round?

**Authors’ response:** We adjusted for the year of survey in our analysis. Added text in lines 522 – 523.

13. In the Discussion, the authors highlight many variables as “relatively important” in XGBoost but not significant in logistic regression. Please explain why this might occur (e.g., nonlinear effects, interactions, or differing sensitivity to correlated predictors).

**Authors’ response:** We have added text in lines 396 – 397.

14. In the discussion of the strengths and limitations of XGBoost, include comments on calibration, and external validation.

**Authors’ response:** We have added text in lines 542– 543.

15. For the imputation process, specify the missRanger settings, number of imputations (single vs. multiple), whether the imputation included the outcome variable, and how imputation uncertainty was handled or propagated.

**Authors’ response:** We have added text in lines 164 – 169.
